# BI-2536 and BI-6727, dual Polo-like kinase/bromodomain inhibitors, effectively reactivate latent HIV-1

**DOI:** 10.1038/s41598-018-21942-5

**Published:** 2018-02-23

**Authors:** Jin Gohda, Kazuo Suzuki, Kai Liu, Xialin Xie, Hiroaki Takeuchi, Jun-ichiro Inoue, Yasushi Kawaguchi, Takaomi Ishida

**Affiliations:** 1 China-Japan Joint Laboratory of Molecular Immunology & Microbiology, Institute of Microbiology, Chinese Academy of Sciences, Beijing, P. R. China; 2 Research Center for Asian Infectious Diseases, The Institute of Medical Science, The University of Tokyo, Tokyo, Japan; 3 Immunovirology Laboratory, St. Vincent’s Center for Applied Medical Research, Darlinghurst, New South Wales Australia; 4 Department of Molecular Virology, Tokyo Medical and Dental University, Tokyo, Japan; 5 Division of Cellular and Molecular Biology, The Institute of Medical Science, The University of Tokyo, Tokyo, Japan; 6 Division of Molecular Virology, The Institute of Medical Science, The University of Tokyo, Tokyo, Japan

## Abstract

HIV-1 latent reservoirs harbouring silenced but replication-competent proviruses are a major obstacle against viral eradication in infected patients. The “shock and kill” strategy aims to reactivate latent provirus with latency reversing agents (LRAs) in the presence of antiretroviral drugs, necessitating the development of effective and efficient LRAs. We screened a chemical library for potential LRAs and identified two dual Polo-like kinase (PLK)/bromodomain inhibitors, BI-2536 and BI-6727 (volasertib), which are currently undergoing clinical trials against various cancers. BI-2536 and BI-6727 significantly reactivated silenced HIV-1 provirus at both the mRNA and protein level in two latently infected model cell lines (ACH2 and U1). BI-2536 dramatically reactivated transcription of latent HIV-1 provirus in peripheral blood mononuclear cells derived from infected patients. Long terminal repeat activation by the inhibitors was associated with bromodomain rather than PLK inhibition. We also found that BI-2536 synergistically activates the latent provirus in combination with SAHA, a histone deacetylase inhibitor, or the non-tumour-promoting phorbol ester prostratin. Our findings strongly suggest that BI-2536 and BI-6727 are potent LRAs for the “shock and kill” HIV-1 eradication strategy.

## Introduction

The use of highly active antiretroviral therapy (HAART) has contributed considerably to extending the life span of patients infected with human immunodeficiency virus type 1 (HIV-1) by preventing acquired immunodeficiency syndrome (AIDS) from developing. Although HAART can reduce viral loads to undetectable levels, HIV-1 is not completely eradicated. A major barrier to viral eradication is the persistence of latently infected reservoirs that can evade both the host antiviral response and HAART^1^. Although a variety of cell types, including T-cells, monocytes, macrophages, and dendritic cells, may form latent reservoirs^2^, resting memory CD4+ T cells are considered the critical latent reservoir^1–3^. Proviruses in the latent reservoir cells are replication-competent but completely or almost completely transcriptionally repressed, due to suppression of HIV-1 long terminal repeat (LTR) promoter activity^4^. However, appropriate cellular stimuli enable the latent reservoir cells to reactivate the provirus and produce infectious viral particles again. Therefore, latent reservoir cells are a major potential source of viral rebound after interruption of the HAART.

The “shock and kill” or “kick and kill” strategy has received much attention as a promising approach to HIV-1 eradication^5,6^. First, latent HIV-1 is “shocked”; in other words, silent proviruses in the latent reservoirs are forcibly reactivated by treatment with latency reversing agents (LRAs) during HAART to prevent susceptible cells from becoming infected with the newly produced viruses. The second step is to “kill” the latent reservoir cells using the viral cytopathic effect and host antiviral immune responses, leading to elimination or reduction of the latent reservoir pool.

To date, many small molecules and agents have been suggested as potential LRAs for the “shock and kill” strategy^6,7^. Based on their functional mechanisms, these agents can be broadly categorized as follows: (i) epigenetic modifiers of the HIV-1 LTR promoter region; (ii) activators of transcriptional factors, such as nuclear factor kappa-light-chain-enhancer of activated B cells (NF-κB) and activator protein 1 (AP-1); and (iii) activators of positive transcription elongation factor (P-TEFb), which is required for RNA polymerase II elongation of HIV-1 mRNA.

Histone deacetylase (HDAC) inhibitors, such as SAHA (vorinostat), efficiently reactivate the latent provirus *in vitro* and *ex vivo* through histone acetylation in or around the HIV-1 LTR, thus changing its epigenetic status^8,9^. Protein kinase C (PKC) agonists, such as non-tumor-promoting phorbol esters prostratin, also strongly reactivate the latent provirus^10,11^. PKC agonists activate transcriptional factors, such as NF-κB and AP-1, which bind to their recognition sites within the LTR and activate viral mRNA transcription^12^.

Bromodomain and extraterminal (BET) bromodomain inhibitors block the binding of bromodomains to acetylated lysine residues. It has been demonstrated that various bromodomain inhibitors, such as JQ1, I-BET, and OTX015, can reactivate the latent HIV-1 provirus^13–16^. For example, bromodomain inhibitors facilitate recruitment of P-TEFb to LTRs by blocking interaction between P-TEFb and a BET protein, bromodomain-containing protein 4 (BRD4)^17–19^. It has been recently shown that a short isoform of BRD4 interacts with a SWI/SNF chromatin-remodelling complex through BRD4 bromodomains and recruits the protein complex to the LTR region, resulting in the inhibition of HIV-1 transcription^20^. JQ1 dissociates the BRD4-SWI/SNF protein complex from the HIV-1 LTR region, which prevents BRD4-mediated suppression of the LTR transcription. Other BRD4-independent reactivation mechanisms have also been suggested with this drug^14^.

Despite clinical trials on the therapeutic ‘shock and kill” potential of several LRAs^7^, none have effectively reduced the size of HIV-1 reservoirs possibly because the responsiveness of the latent provirus to each LRA may depend on the reservoir cell type. In addition, the chromosomal location of the integrated provirus may influence the responsiveness of the infected cells to LRAs^21^. Therefore, development of new LRAs or combination therapy using multiple LRAs to efficiently reactivate latent HIV-1 provirus is a priority

We report here that two Polo-like kinase (PLK) inhibitors, BI-2536 and BI-6727 (also known as volasertib), effectively reactivate latent HIV-1. BI-2536 was identified during small-molecule screening for kinase inhibitors of PLK1^22^, a member of the PLK family that consists of four serine/threonine protein kinases (PLK1, PLK2, PLK3 and PLK4). BI-6727 was developed by modifying the chemical structure of BI-2536^23^. PLK1 contributes to the G2/M phase of cell cycle^24^ and is also associated with tumorgenesis^25,26^. Therefore, inhibitors of PLK1 kinase activity are expected to be effective anticancer agents. In fact, BI-2536 and BI-6727 have been tested in phase I/II and I/II/III clinical trials, respectively, for treatment of various cancers. Moreover, it has recently been demonstrated that BI-2536 and BI-6727 also inhibit bromodomain function, hypothetically making them potent LRAs^27^. In this study, we evaluated the potential of BI-2536 and BI-6727 as potential LRAs in the “shock and kill” HIV-1 eradication strategy.

## Results

### Development of myeloid-linage derived cell models for LRA screening

**Figure 1:**
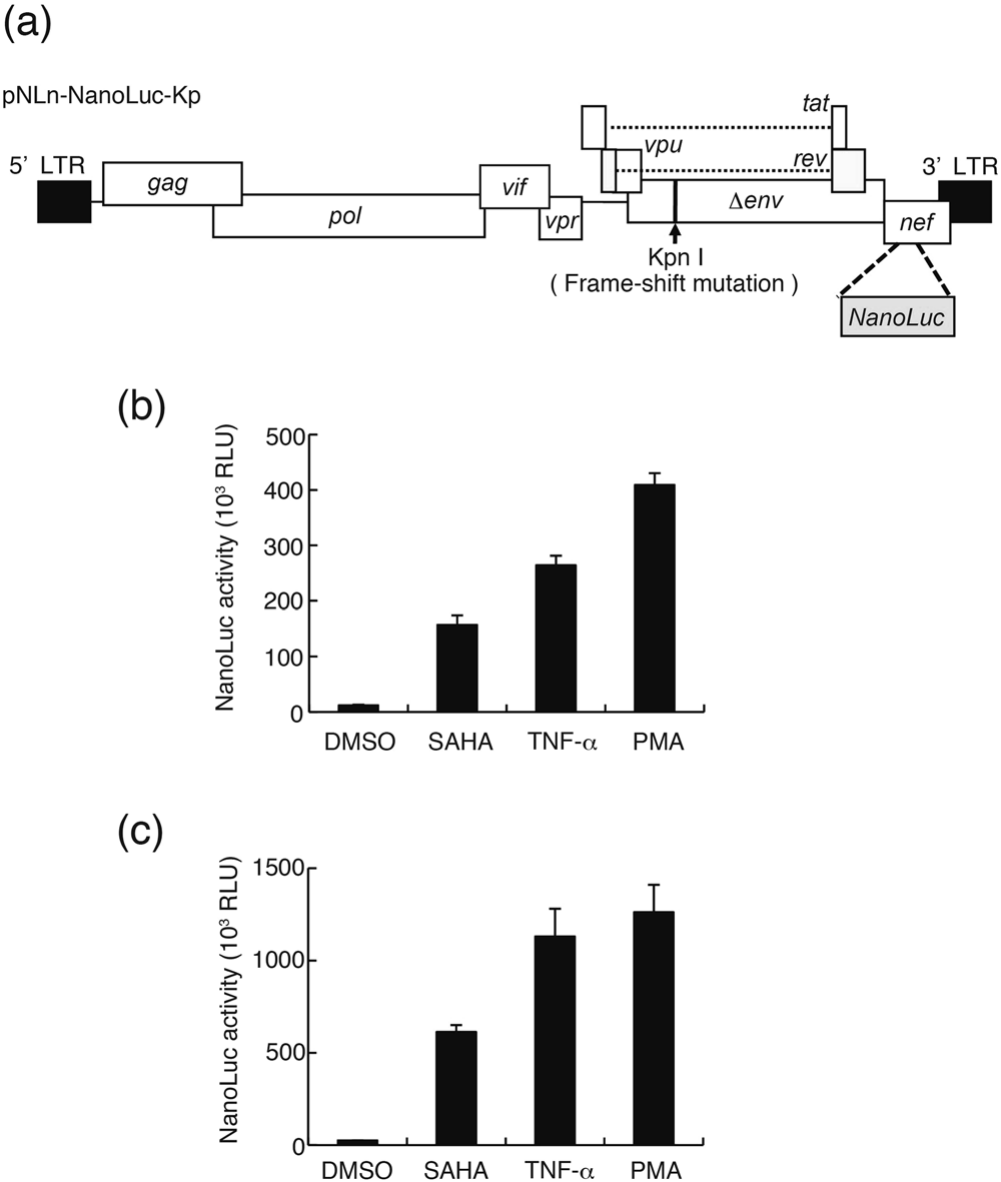
SAHA, tumor necrosis factor alpha (TNF-α), and phorbo-12-myristate-13-acetate (PMA) induce NanoLuc activity in THP-1-NanoLuc clones. (**a**) Schematic structure of an HIV-1 vector designated pNLn-NanoLuc-Kp is shown. The vector has a frame-shift mutation at the KpnI site in the *env* gene to avoid expression of the viral envelop protein. Part of the *nef* gene is replaced with NanoLuc that functions as a reporter gene. The VSV-G pseudotyped virus was produced by co-transfection into HEK293FT cells with pNLn-NanoLuc-Kp and a VSV-G expression vector. After THP-1 cells were infected with the virus, the infected clones were obtained by a limiting dilution method. (**b**) #95 and (**c**) #225 THP-1-NanoLuc clone cells were stimulated with 10 μM SAHA, 10 ng/mL TNF-α, or 10 ng/mL PMA for 24 hours. The cells were harvested and lysed. NanoLuc activity of the cell lysate was determined. Each value is shown as a mean ± standard deviation (SD) for triplicate samples.

J-Lat cells derived from Jurkat T cell lines have been widely used as HIV-1 latent T cell models, contributing to clarifying the mechanisms of viral latency and screening of LRAs^28^. Although myeloid-linage cells, such as monocytes and macrophages, are also an important latent reservoir, there are no established myeloid-lineage-derived latent cell models suitable for chemical library screening of LRAs. Therefore, we attempted to establish myeloid-linage latent model cells harboring a reporter gene for chemical library screening to cover other cell-types than T cells. We constructed an HIV-1-based viral vector, pNLn-NanoLuc-Kp, with a frame-shift mutation in the *env* region of the NL4–3 laboratory strain of HIV-1 and containing a NanoLuc gene as a reporter in the *nef* region (Fig. 1a). THP-1 cells, a human monocyte-linage cell line, were infected with the vesicular stomatitis virus G (VSV-G) pseudotyped NLn-NanoLuc-Kp. After infection, we obtained the infected cell clones by a limiting dilution method. We selected the two clones, #95 and #225, with much lower NanoLuc activity compared to the other infected cell clones. NanoLuc activity in both clones increased significantly in response to SAHA, tumor necrosis factor alpha (TNF-α), and phorbol-12-myristate-13-acetate (PMA) which are the well-known activating agents of HIV-1 gene expression (Fig. 1b,c). Therefore, we decided to use these clones to screen a chemical library for compounds that activated HIV-1 gene expression.

### BI-2536 and BI-6727 reactivate HIV-1 provirus in different latently infected model cells

**Figure 2:**
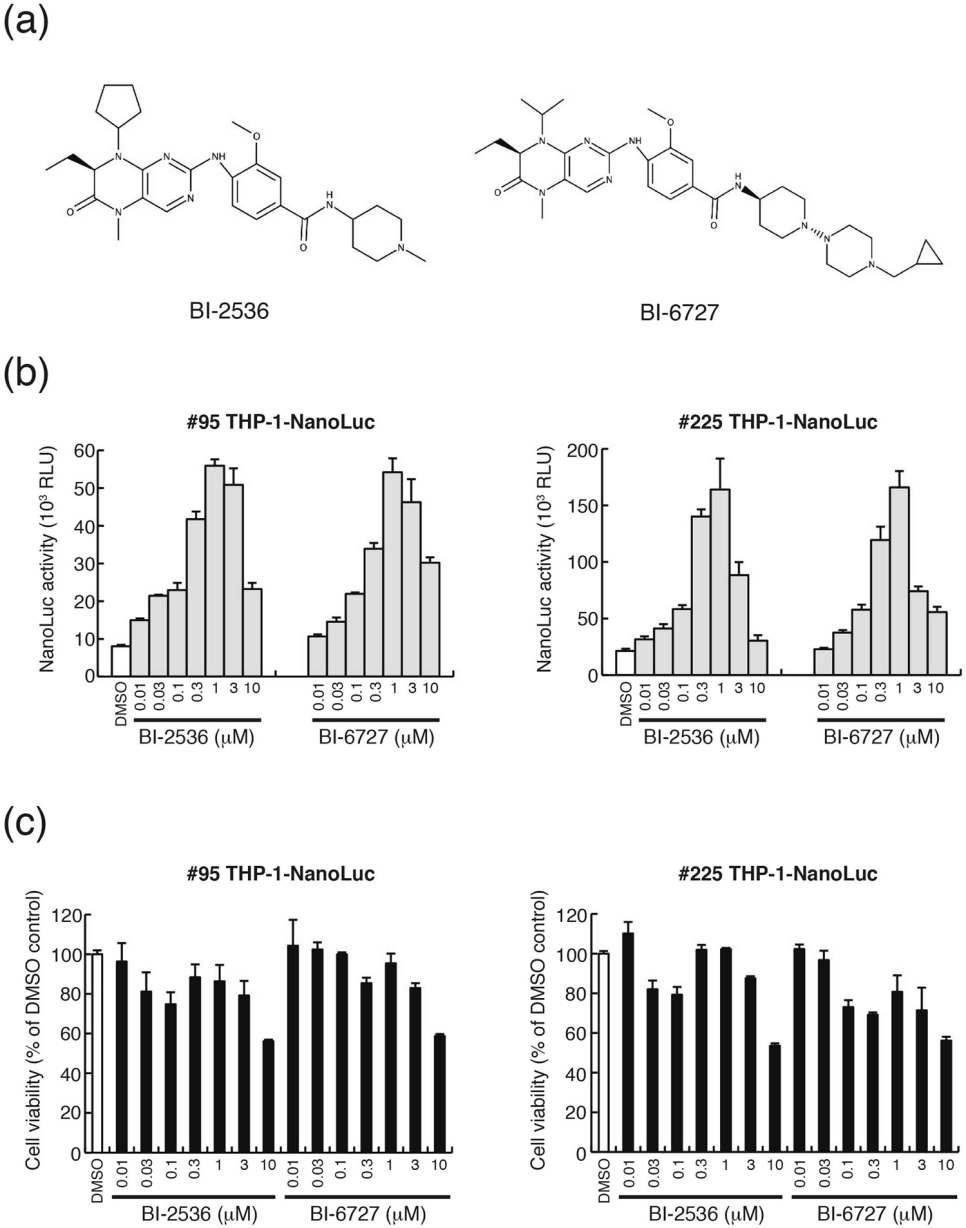
BI-2536 and BI-6727 induce NanoLuc activity in THP-1-NanoLuc clones. (**a**) The structures of BI-2536 and BI-6727. (**b**) #95 THP-1-NanoLuc clone and #225 clone cells were cultured in the absence (0.1% dimethyl sulfoxide [DMSO]) or presence of BI-2536 or BI-6727 at the indicated concentrations for 24 hours. The cells were then harvested and lysed. NanoLuc activity of the cell lysates was determined. Each value is shown as a mean ± standard deviation (SD) of triplicate samples. (**c**) #95 and #225 THP-1-NanoLuc clone cells were cultured in 0.1% DMSO or in the presence of BI-2536 or BI-6727 at the indicated concentrations for 24 hours. Cell viability was measured by a WST-8 assay and shown as a percentage of that of DMSO control cells. Each value is shown as a mean ± standard deviation (SD) for triplicate samples.

We screened a commercial kinase inhibitor library consisting of 378 inhibitors and identified two inhibitors of PLK family kinases, BI-2536 and BI-6727 that were efficient activators of HIV-1 LTR transcription. BI-2536 and BI-6727 have chemical structures similar to dihydropteridinone derivatives (Fig. 2a). BI-2536 and BI-6727 significantly induced NanoLuc expression in a dose-dependent manner in #95 and #225 THP-1- NanoLuc clones (Fig. 2b). Both inhibitors displayed maximal induction at a dose of 1 μM. BI-2536 and BI-6727 were slight cytotoxic to THP- NanoLuc clones up to a dose of 3 μM (cell viabilities of >80%), but this effect was not significant (Fig. 2c). However, BI-2536 and BI-6727 induced severe cell death at concentrations over 0.03 μM and 0.1 μM, respectively (Supplementary Fig. 1), consistent with previous reports^22,23^.

**Figure 3:**
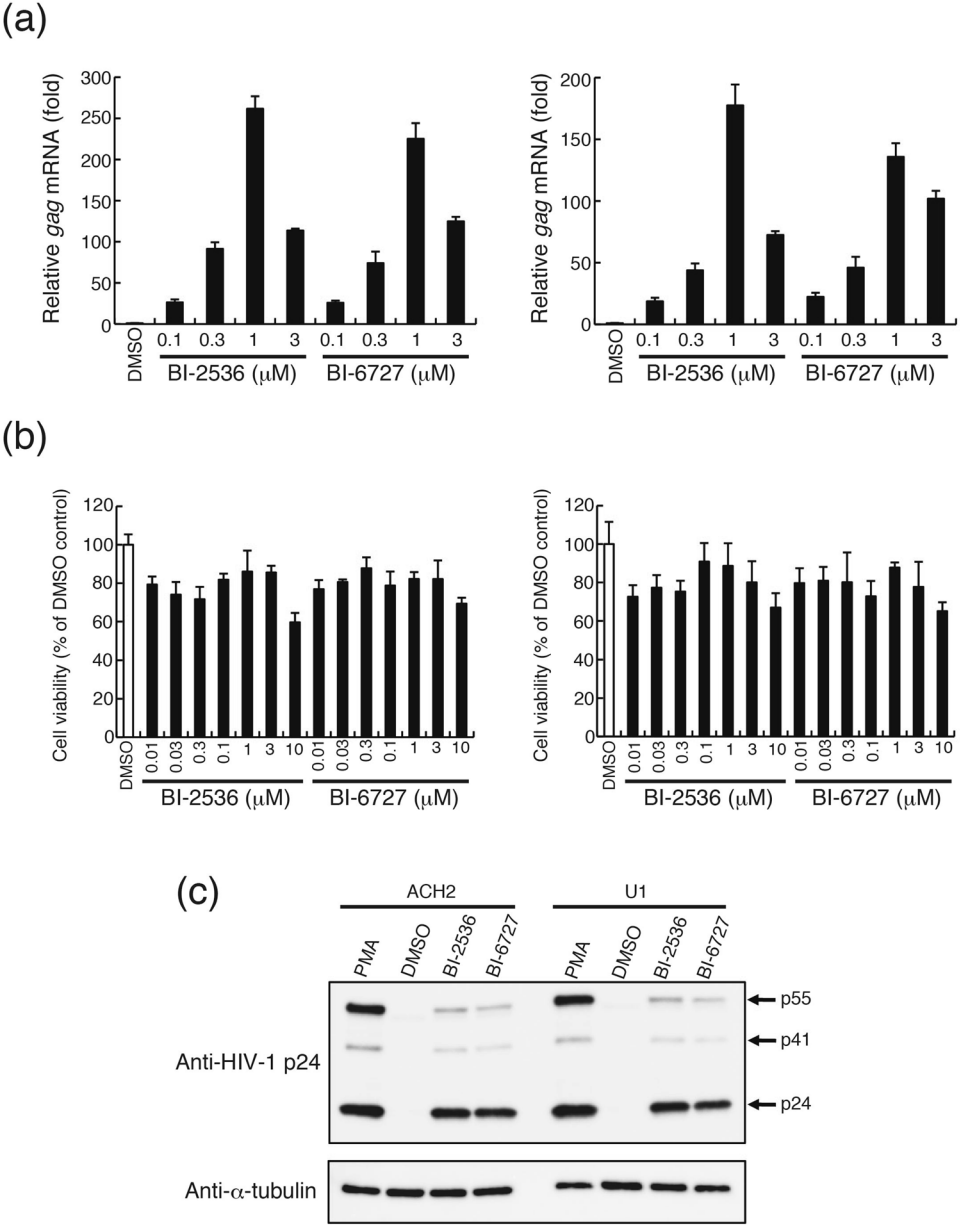
BI-2536 and BI-6727 reactivate HIV-1 provirus in the latently infected model cells. (**a**) ACH2 and U1 cells were cultured in the presence of 0.1% DMSO, BI-2536 or BI-6727 at the indicated concentrations for 16 hours. HIV-1 *gag* mRNA expression was measured by quantitative RT-PCR. Fold induction of *gag* mRNA normalized by *gapdh* mRNA expression was determined and compared with DMSO-treated cells. Each value is shown as a mean ± SD of triplicate determinations. (**b**) ACH2 and U1 cells were cultured in 0.1% DMSO only or in the presence of BI-2536 or BI-6727 at the indicated concentrations for 24 hours. Cell viability was measured by a WST-8 assay and shown as a percentage of that of DMSO control cells. Each value is shown as a mean ± standard deviation (SD) for triplicate samples. (**c**) ACH2 and U1 cells were incubated in the presence of 0.1% DMSO, 10 ng/mL PMA, 1 μM BI-2536, or 1 μM BI-6727 for 24 hours. Cell lysates were immunoblotted with anti-HIV-1 p24 and anti-α-tubulin antibodies. The arrows indicate bands of viral p24, p41, and p55. The cropped images of the blots are shown. The uncropped images are shown in Supplementary Fig. 2.

Next, we examined whether BI-2536 and BI-6727 reactivate the HIV-1 provirus in latently infected model cells. T-cell-derived ACH2 and monocyte-derived U1 cells have severely silenced HIV-1 proviruses in their genomes^29,30^. These cells were treated with BI-2536 or BI-6727 for 16 hours and expression levels of HIV-1 *gag* mRNA detected by quantitative real-time RT-PCR. BI-2536 and BI-6727 dramatically induced *gag* mRNA expression in a dose-dependent manner in ACH2 and U1 cells (Fig. 3a). The highest induction levels were obtained with 1 μM BI-2536 (261-fold in ACH2 cells and 177-fold in U1 cells) and 1 μM BI-6727 (225-fold in ACH2 cells and 136-fold in U1 cells). Treatment with BI-2536 or BI-6727 for 16 hours resulted in a slight, insignificant decrease in cell viabilities of ACH2 and U1 cells up to 3 μM (cell viabilities of >70%) (Fig. 3b). However, similar to THP- NanoLuc clones, BI-2536 and BI-6727 exhibited induction of severe cell death at concentrations over 0.03 μM and 0.1 μM, respectively (Supplementary Fig. 1).

To test whether HIV-1 viral protein is produced after treatment with BI-2536 or BI-6727, we used western blotting to detect HIV-1 Gag p24 protein expression levels (Fig. 3c and Supplementary Fig. 1). Gag p24 protein was not detectable in DMSO-treated control ACH2 and U1 cells. Treatment with PMA as positive control induced p24 and its precursor proteins, p41 and p55 in both cell lines. Moreover, treatment with 1 μM BI-2536 or 1 μM BI-6727 also significantly induced expression of viral proteins. Taken together, these results indicate that BI-2536 and BI-6727 reactivate HIV-1 provirus at both mRNA and protein levels in latently infected cell lines.

### BI-2536 reactivates the latently infected HIV-1 provirus in infected patient samples

**Figure 4:**
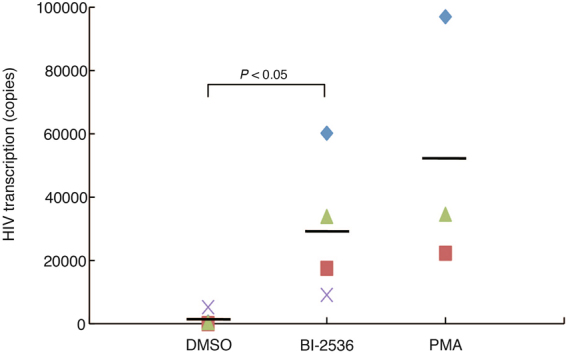
BI-2536 reactivates latent HIV-1 provirus in primary cells from HIV-1 infected patients. Peripheral blood mononuclear cells (PBMCs) derived from four different HIV-1 patients were treated with dimethyl sulfoxide (DMSO) or with 1 μM BI-2536. PBMCs from three patients were treated with 10 ng/mL phorbol-12-myristate-13-acetate (PMA) for 16 hours. Total RNA was prepared from the cells. *Gag* mRNA expression was measured by quantitative RT-PCR. Viral copy number per one million cells was shown as the mean of triplicate determination. The horizontal lines show the mean of the samples treated with each regent. *P*-values were determined using the Mann-Whitney *U* test for nonparametric data. *P*-values are significant if less than 0.05.

Next, we examined whether BI-2536 could activate HIV-1 gene expression in primary cells from HIV-1-infected patients. Peripheral blood mononuclear cells (PBMCs) were obtained from four HIV-1-infected patients under HAART who had plasma viral RNA loads of less than 20 copies. PBMCs from all four patients were treated with BI-2536 for 16 hours and viral RNA cell copy numbers were determined by absolute quantitative RT-PCR. PBMCs from three patients were also treated with PMA as positive control. Viral copy numbers of all four patient samples increased in response to BI-2536 (Fig. 4). There was a significant difference in copy number (*p* values < 0.05) between the BI-2536-treated group and DMSO-control group on the Mann-Whitney test. These results strongly suggest that BI-2536 reactivates HIV-1 gene expression in PBMCs from infected patients.

### BI-2536 and BI-6727 activate HIV-1 LTR transcription through bromodomain rather than PLK inhibition

**Figure 5:**
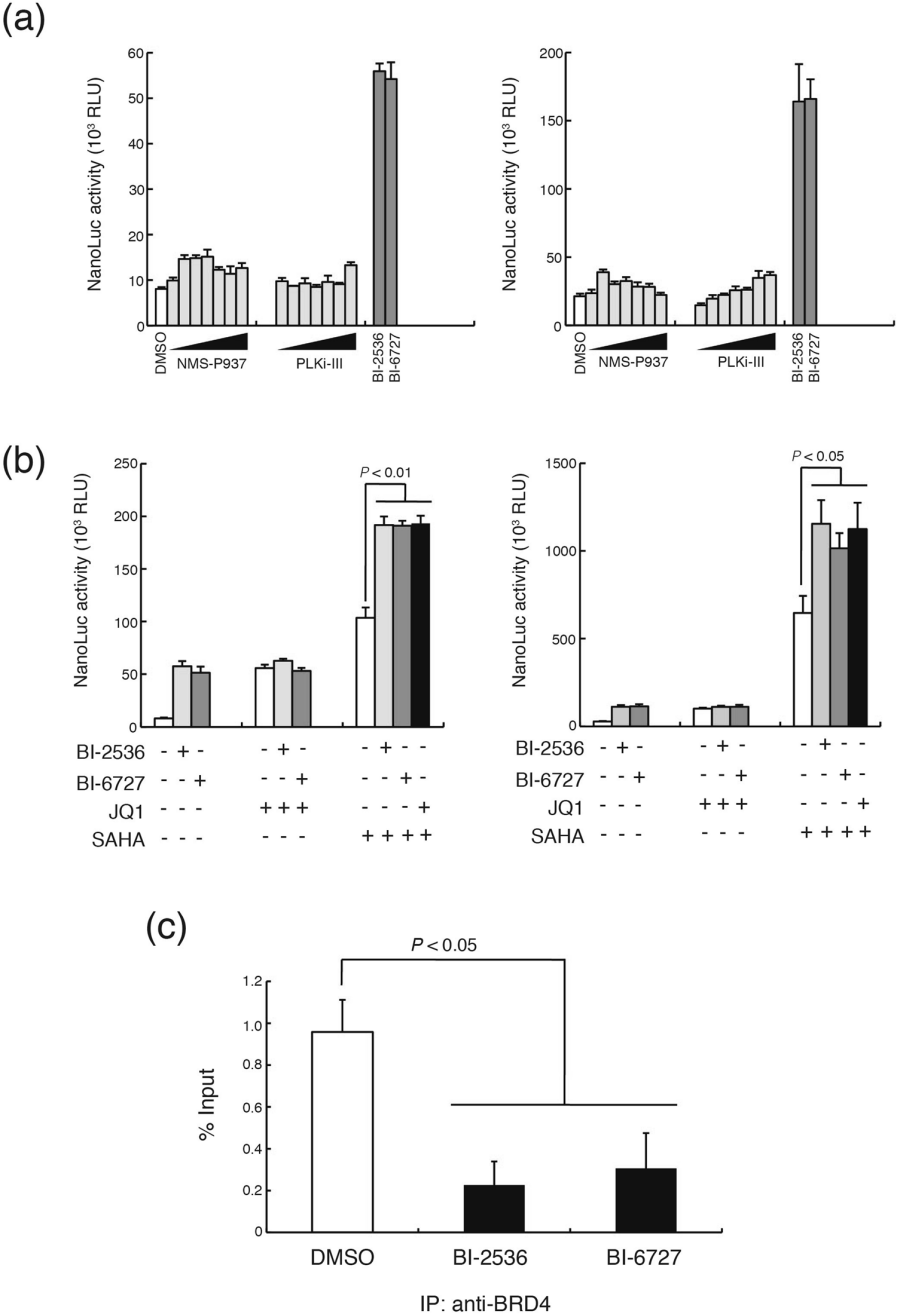
BI-2536 and BI-6727 activate HIV-1 LTR transcription probably through bromodomain inhibition rather than PLK inhibition. (**a**) #95 and #225 THP-1-NanoLuc clones were cultured in 0.1% dimethyl sulfoxide (DMSO) in the absence or presence of NMS-P937 or PLKi-III at 0.01, 0.03, 0.1, 0.3, 1, 3, and 10 μM for 24 hours. The clones were also cultured with 1 μM BI-2536 or 1 μM BI-6727. After the clone cells were harvested and lysed, the NanoLuc activity of cell lysates was determined. Each value is shown as a mean ± standard deviation (SD) of triplicate samples. (**b**) #95 and #225 THP-1-NanoLuc clones were cultured without (−) or with (+) 1 μM BI-2536 or 1 μM BI-6727 in the absence (−) or presence (+) of 1 μM JQ1 or 10 μM SAHA for 24 hours. After the cells were harvested and lysed, NanoLuc activity of the cell lysates was determined. Each value is shown as a mean ± SD of triplicate samples. *P*-values were determined using Student’s t-test. *P*-values are significant if less than 0.05. (**c**) #95 THP-1-NanoLuc clone cells were treated with 0.1% DMSO, 1 μM BI-2536 or 1 μM BI-6727 for 24 hours and subjected to ChIP assay. The level of BRD4 protein associated with the HIV-1 LTR promoter was determined. ChIP DNA signals were normalized by those of input DNA. Each value is shown as a mean ± SD of triplicate samples. *P*-values were determined using Student’s t-test. *P*-values are significant if less than 0.05.

Although BI-2536 and BI-6727 were initially developed as highly selective and potent small molecule inhibitors of PLK1, both inhibitors block kinase activities of other PLK family members at high concentrations. Half maximal inhibitory concentration (IC_50_) values of BI-2536 against PLK1, PLK2, and PLK3 in cell-free kinase assays are 0.83 nM, 3.5 nM, and 9.0 nM, respectively^22^. BI-6727 inhibits PLK1, PLK2, and PLK3 at an IC50 of 0.87 nM, 5 nM, and 56 nM, respectively^23^. To examine whether BI-2536 and BI-6727 reactivate latently infected HIV-1 provirus by inhibiting PLKs, we tested a selective PLK1 inhibitor, NMS-P937 and a pan-PLK inhibitor, PLK inhibitor III (PLKi-III)^31–33^. However, unlike BI-2536 and BI-6727, NMS-P937 and PLKi-III did not significantly affect the NanoLuc activities up to 10 μM in #95 and #225 THP-1-NanoLuc clones (Fig. 5a). These results suggest that BI-2536 and BI-6727 probably reactivate the latently infected HIV-1 independently of PLK inhibition.

BI-2536 and BI-6727 have recently been reported to inhibit BET bromodomain proteins as well as PLKs. BI-2536 and BI-6727 bind to BRD4 with a dissociation constant (*K*_*d*_) of 37 nM and 79 nM, respectively^27^. It has been also shown that BI-2536 strongly inhibits BRD4-dependent expression of c-Myc in multiple myeloma cells in a similarly way to the bromodomain inhibitor JQ1^27^. To examine whether bromodomain inhibition by BI-2536 and BI-6727 is involved in the HIV-1 LTR activation, we investigated the effect of combination treatment with BI inhibitors and JQ1 or an HDAC inhibitor, SAHA. When #95 and #225 THP-1-NanoLuc clones were treated with 1 μM BI-2536 or 1 μM BI-6727 together with JQ1 at a concentration of 1 μM for maximal NanoLuc activation, there was no difference in NanoLuc induction between cells treated with BI-2536, BI-6727, or JQ1 alone, or those treated in combination with JQ1 (Fig. 5b). In contrast, NanoLuc induction was enhanced following co-treatment with BI-2536 or BI-6727 and SAHA compared to treatment with each inhibitor alone. These results suggest that JQ1 and both BI inhibitors may target the same biological pathways of activation of HIV-1 gene expression, and that SAHA and the BI inhibitors might induce HIV-1 gene expression via distinct mechanisms.

**Figure 6:**
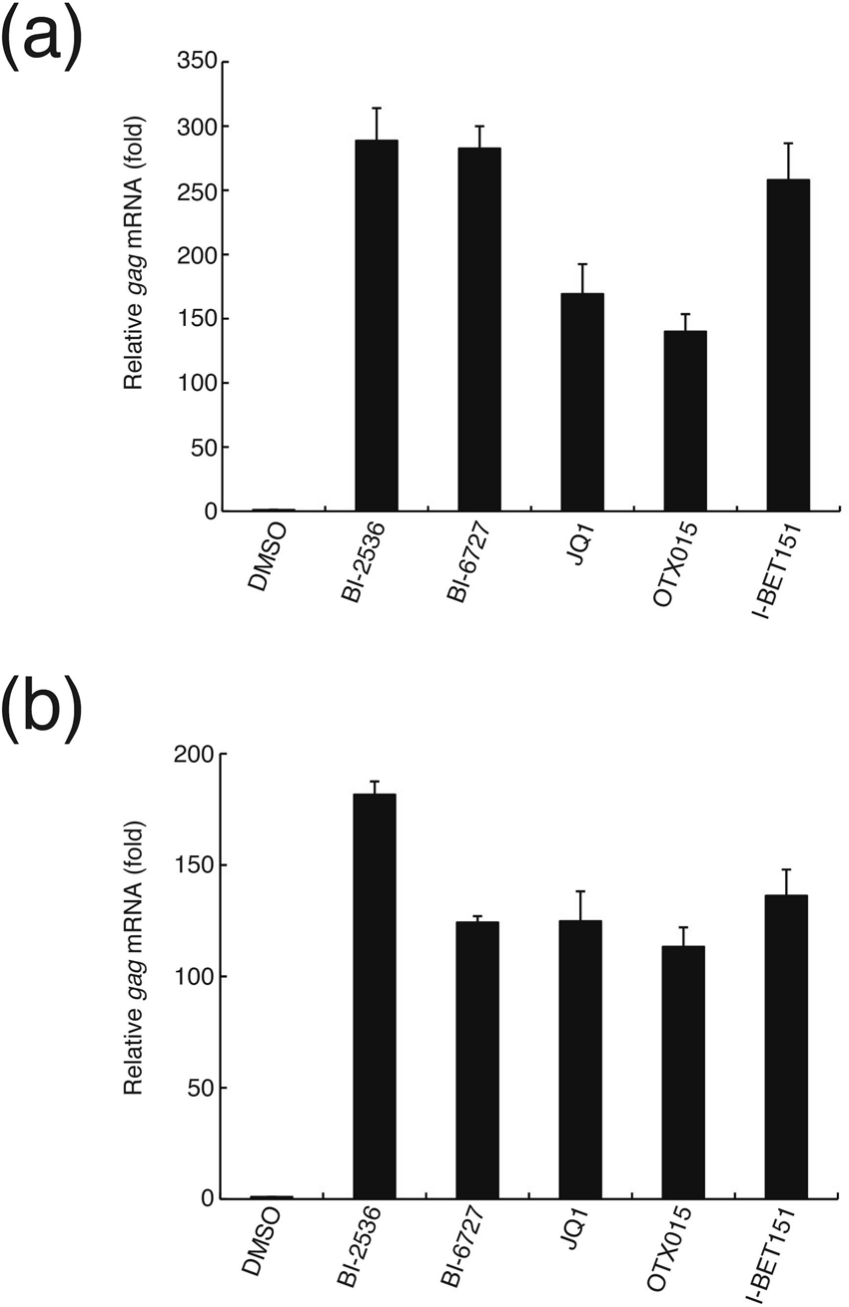
BI-2536, BI-6727, and various BET inhibitors reactivate latent HIV-1 provirus in ACH2 and U1 cells. (**a**) ACH2 and (**b**) U1 cells were cultured in 0.1% dimethyl sulfoxide (DMSO) only or in the presence of 1μM BI-2536, 1 μM BI-6727, 1 μM JQ1, 3 μM OTX015, or 10 μM I-BET151 for 16 hours. HIV-1 *gag* mRNA expression was measured by quantitative RT-PCR. Fold induction of *gag* mRNA normalized by *gapdh* mRNA expression was compared with that in DMSO-treated cells. Each value is shown as a mean ± standard deviation (SD) for triplicate samples.

It has been previously demonstrated that the BET bromodomain inhibitor JQ1 dissociates BRD4 from the HIV-1 LTR promoter^19,20^. Therefore, we examined the effect of BI-2536 and BI-6727 on BRD4 recruitment to the HIV-1 LTR by the chromatin immunoprecipitation (ChIP) assay. ChIP analysis showed that BI2536 or BI-6727 significantly reduced the level of BRD4 protein associated with the HIV-1 LTR region (Fig. 5c). Furthermore, BI-2536 and BI-6727 reactivated latent HIV-1 provirus at the same or a slightly higher level as other BET-bromodomain inhibitors, such as JQ1, OTX015, and I-BET151 (Fig. 6). Taken together, these results suggest that BI-2536 and BI-6727 probably reactivate the latent HIV-1 provirus by inhibiting the bromodomain rather than PLK kinase.

### Latent HIV-1 proviruses in primary cells from infected patients have different responsiveness to BI-2536 and JQ1

**Figure 7:**
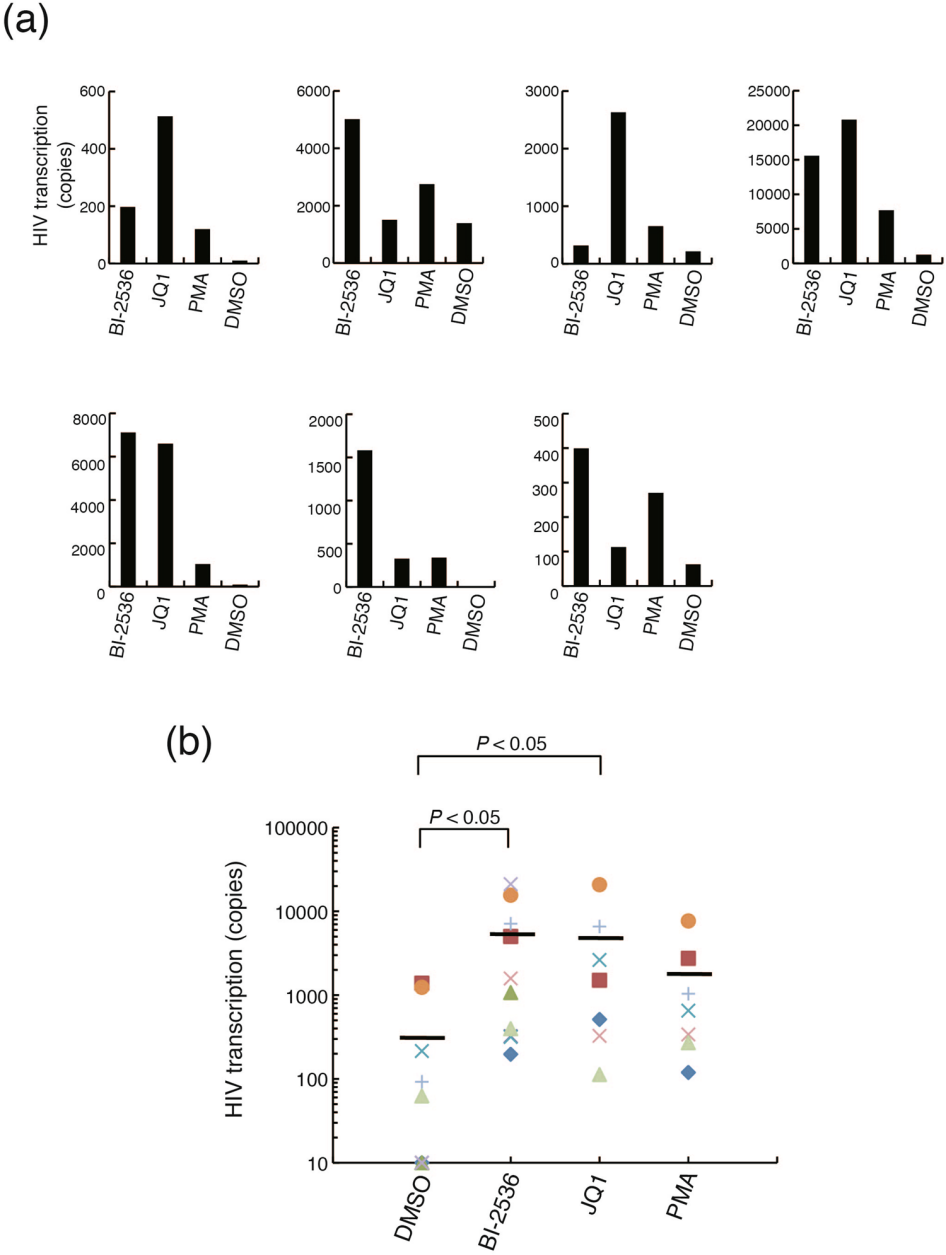
Latent HIV-1 proviruses in primary cells from infected patients have different responsiveness to BI-2536 and JQ1. (**a**) Peripheral blood mononuclear cells (PBMCs) derived from seven different HIV-1 patients were expanded in the presence of IL-2 and PHA. The cells were then cultured in 0.1% dimethyl sulfoxide (DMSO) and untreated or treated with 1 μM BI-2536, 1 μM JQ1, or 10 ng/mL phorbol-12-myristate-13-acetate (PMA) for 16 hours. Total RNA was prepared from the cells. *Gag* mRNA expression was measured by quantitative RT-PCR. Viral copy number per one million cells was shown as the mean of triplicate determination. (**b**) Statistical analysis was conducted on the data shown in (**a**). The horizontal lines show the mean of the samples treated with each regent. *P*-values were determined using the Mann-Whitney *U* test for nonparametric data. *P*-values are significant if less than 0.05.

Next, we compared the ability of BI-2536 and JQ1 to reactivate latent HIV-1 provirus in primary cells from infected patients. PBMCs derived from seven HIV-1-infected patients were expanded with IL-2 and PHA to obtain enough cells for mRNA extraction from the cells stimulated with several reagents, since we only had a small volume of the patient blood samples. Viral copy numbers in the IL-2/PHA- stimulated PBMCs were determined after treatment with BI-2536, JQ1, or PMA as positive control, for 16 hours. In all samples, there was a significant increase in viral copy number in response to BI-2536 and JQ1 (Fig. 7a and b). However, the responsiveness of the cells to each reagent was different among the patient samples (Fig. 7a). Interestingly, BI-2536 reactivated the provirus more strongly than JQ1 in three patient samples (Patient 150, 157, and 159). In contrast, the effect of JQ1 was stronger than that of BI-2536 in two patient samples (Patient 149 and 153), while there was no significant difference between the induction levels of two samples (Patent 154 and 156) treated with BI-2536 and JQ1. These results suggest that BI-2536 is a better alternative for reactivation of latently infected HIV-1 compared to other BET-bromodomain inhibitors, such as JQ1.

### BI-2536 synergistically reactivates the latent provirus in combination with other LRAs

**Figure 8:**
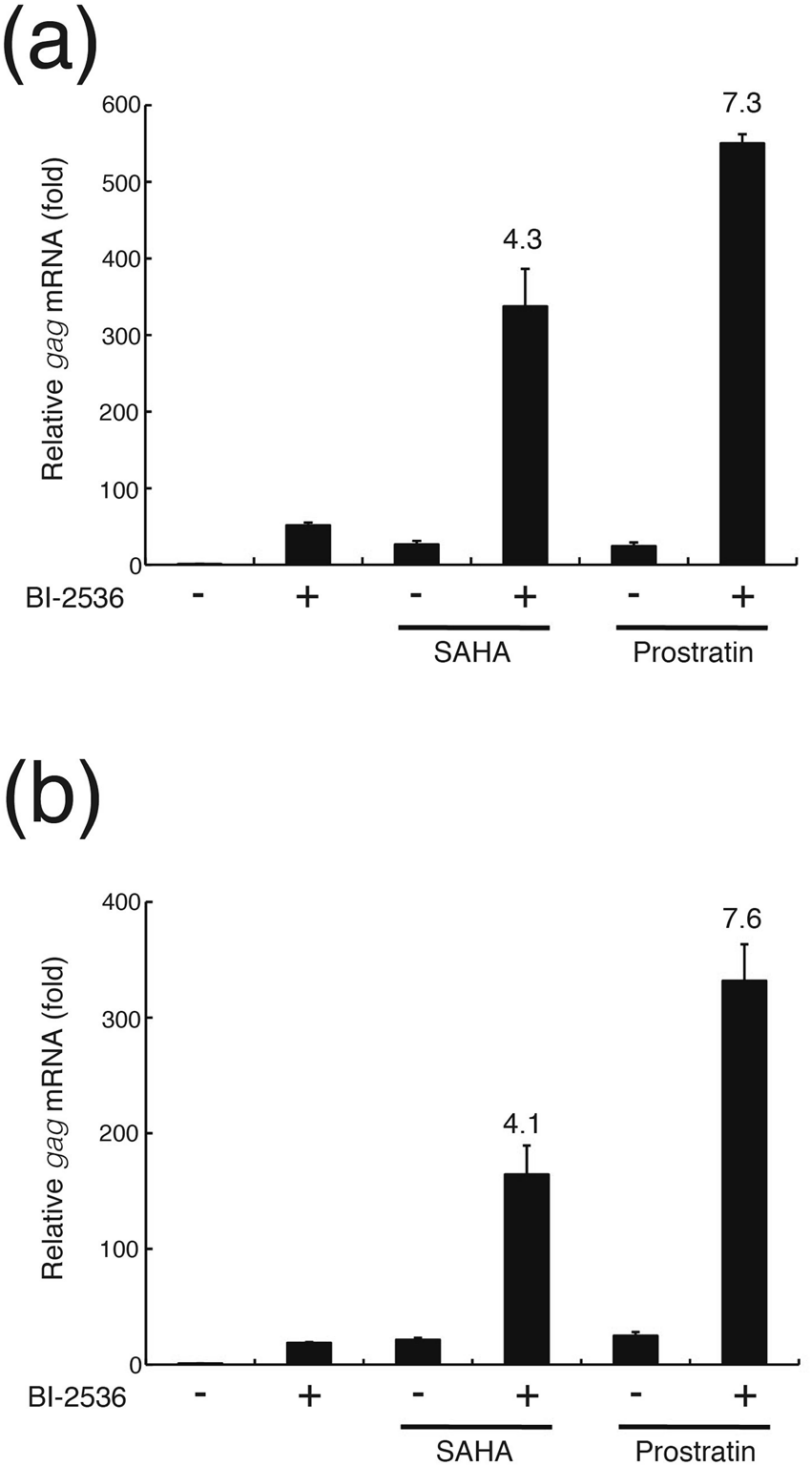
BI-2536 synergistically reactivates latent HIV-1 provirus with SAHA or prostratin in ACH2 and U1 cells. (**a**) ACH2 and (**b**) U1 cells were treated without (−) or with (+) 0.1 μM BI-2536 in combination with 1 μM SAHA or 0.25 μM prostratin for 16 hours. HIV-1 *gag* mRNA expression was measured by quantitative RT-PCR. Fold induction of *gag* mRNA normalized by *gapdh* mRNA expression was determined compared with untreated cells. Each value is shown as a mean ± standard deviation (SD) of triplicate determinations. The values of fold synergy are shown above the bars representing BI-2536 + SAHA and BI-2536 + prostratin. The fold synergy was determined by dividing the fold induction observed in combination treatment with the two inhibitors by the sum of the fold inductions observed in treatment with each of the inhibitors alone.

Lastly, we evaluated the therapeutic potential of BI-2536 to reactivate latent HIV-1 in combination with other LRAs, an HDAC inhibitor or a PKC agonist. Treatment with SAHA (1 μM) or a PKC agonist prostratin (0.25 μM) induced *gag* mRNA expression at low levels in ACH2 cells (24-fold and 27-fold, respectively) and U1 cells (21-fold and 25-fold, respectively) (Fig. 8). However, treatment with BI-2536 (0.1 μM) in combination with SAHA (1 μM) or prostratin (0.25 μM) induced expression 337- and 550-fold in ACH2 cells, and 164- and 332-fold in U1 cells, respectively. The increase in expression for BI-2536 alone was 50-fold in ACH2 cells and 19-fold in U1 cells. We observed that SAHA and prostratin treatment induced 4.3- and 7.3-fold synergistic effect in ACH2 cells, respectively, and 4.1- and 7.6-fold synergistic effect in U1 cells, respectively, when combined with BI-2536. Furthermore, a synergistic reactivation of latent HIV-1 was also observed in PBMCs from one of two independent patients (Patient 201), while BI-2536 enhanced SAHA-induced reactivation of latent HIV-1 in PBMCs from the other patient (Patient 198) (Supplementary Fig. 3). These results indicate that BI-2536 reactivates the latent HIV-1 provirus synergistically with SAHA or prostratin.

## Discussion

The dual PLK/bromodomain inhibitors BI-2536 and BI-6727 have been undergoing clinical trials as anti-cancer agents. In this study, we showed that BI-2536 and BI-6727 effectively reactivate latent HIV-1 in model cell lines and primary cells from infected patients. We postulate that BI-2536 and BI-6727 are clinical LRA candidates for the “shock and kill” HIV-1 eradication strategy, based on the following factors.

Firstly, the effective concentration ranges of BI-2536 and BI-6727 are clinically acceptable. According to anticancer-therapy clinical test data for BI-2536 and BI-6727, the maximum plasmatic concentrations (C_max_) of BI-2536 and BI-6727 are both approximately 1.6 μM when administered at the maximum tolerated dose (MTD) (BI-2536, 200 mg; BI-6727, 400 mg)^34,35^. Our data reveal that both BI-2536 and BI-6727 reactivate the HIV-1 provirus in latent infected cells at a concentration range of 0.3 to 1μM, with maximum reactivation at 1 μM. Importantly, treatment with BI-2536 at a dose of 1 μM significantly reactivated the latently infected provirus in PMBCs from HIV-1 infected patients. These concentrations are apparently lower than the C_max_ values, suggesting BI-2536 and BI-6727 may be acceptable for clinical therapy.

Secondly, BI-2536 could be a useful potent drug to efficiently reactivate latent proviruses that are less sensitive to other bromodomain inhibitors, such as JQ1, since the responsiveness to BI-2536 and JQ-1 varied between patient samples. Although the causes of the differences in responsiveness are not clear, the following possible mechanisms may be involved. Cell types or subtypes contributing to HIV-1 latency or ratios of each of them to the total number of latently infected cells may be different among HIV-1 infected patients, resulting in a divergence in drug sensitivity among the samples; the difference in drug responsiveness may be due to different epigenetic modification of the integrated provirus among infected cells, as it has been reported that the epigenetic environment at the site of chromosomal integration significantly affects responsiveness to LRAs^21^. If this is the case, different bromodomain proteins may be involved in HIV-1 latency in each patient. Furthermore, there is a possibility that PLK inhibition by BI-2536 may contribute to reactivation of the latent provirus in certain patients. Further studies on these possible mechanisms are needed.

Lastly, BI inhibitors could be used for combination therapy, with different types of LRA. To date, no LRAs have successfully reduced latent HIV-1 pools in the infected patients. Combination therapy using multiple LRAs has been gathering attention, because combinations of dugs can be expected to be more effective and have fewer side effects than single LRAs^6,15,36,37^. We found a synergistic reactivation of the latent provirus even at one-tenth of the most effective dose (1 μM) of BI-2536 in combination with a HDAC inhibitor, SAHA or a PKC agonist, prostratin, suggesting that BI-2536 could be a potent drug to purge latent reservoirs as part of combination therapy. However, it is not yet known which combination of LRA/BI inhibitors would reactivate the latent provirus most efficiently. Further investigation is therefore needed.

We conclude that bromodomain, rather than PLK, inhibition is mainly responsible for reactivation of the latent provirus by BI-2536 and BI-6727, based on the following findings: (i) selective PLK1 and pan-PLK inhibitors did not activate HIV-1 LTR in THP-1-NanoLuc cells, (ii) BI-2536 and BI-6727 enhanced LTR activation by an HDAC inhibitor, SAHA, but not a bromodomain inhibitor, JQ1, (iii) BI-2536 and BI-6727 dissociates BRD4 from the HIV-1 LTR region, (iv) BI-2536 and BI-6727 reactivated latent HIV-1 in ACH2 and U1 cells almost as efficiently as several well-characterized bromodomain inhibitors. However, we cannot rule out the possibility that PLK was involved in inhibiting latent provirus reactivation by BI-2536 and BI-6727. A recent study has reported that PLK1 phosphorylates P-TEFb and inhibits P-TEFb-dependent HIV-1 LTR transcription^38^. Although it is unclear whether the inhibition of P-TEFb by PLK1 is involved in HIV-1 latency, upregulation of P-TEFb activity by BI-2536- and BI-6727-induced PLK1 inhibition may have made some contribution to provirus reactivation in the latently infected cells. In addition, G2/M cell cycle arrest following PLK1 inhibition may increase LTR promoter activity, since it has been reported that LTR transcription is at the highest level in the G2 phase of the cell cycle^39^. Further elucidation of the molecular mechanisms underlying reactivation of latent HIV-1 by BI-2536 and BI-6727 is needed.

In conclusion, our results suggest that the dual PLK/bromodomain inhibitors BI-2536 and BI-6727 have therapeutic potential as LRAs to effectively reactivate latent HIV-1, thus eliminating latent reservoirs, although the precise mechanism of latent provirus reactivation by the inhibitors needs to be clarified. Most importantly, it is hoped that BI-2536 and BI6727 will undergo clinical trials as LRA drugs in the “shock and kill” HIV-1 eradication approach.

## Methods

### Cell culture and regents

THP-1, THP-1-NanoLuc-clones, ACH2, and U1 cells were maintained at 37 °C in 5% CO_2_ in Gibco RPMI1640 Medium (Thermo Fisher Scientific) supplemented with 10% heat-inactivated fetal bovine serum. HEK293FT cells were maintained in Dulbecco’s modified Eagle’s medium (Thermo Fisher Scientific) supplemented with 10% heat-inactivated fetal bovine serum. BI-2536, BI-6797, SAHA (vorinostat), NMS-P937, JQ1, OTX015, and I-BET151 were purchased from Selleckchem. Phorbol-12-myristate-13-acetate (PMA) and prostratin were obtained from Sigma-Aldrich. Human tumor necrosis factor-alpha (hTNF-α) and PLK inhibitor III were purchased from PeproTech and Calbiochem, respectively. Kinase Inhibitor Library (L1200) with 378 kinase inhibitors was obtained from Selleckchem.

### Establishment of THP-1-NanoLuc clones and chemical library screening

An HIV-1-based virus vector, pNLn-NanoLuc-Kp^40^, was constructed by replacing an enhanced green fluorescent protein gene in pNLnGFP-Kp with a NanoLuc gene fragment using an In-Fusion HD Cloning Kit (Takara Bio). To prepare the virus, HEK293FT cells were co-transfected with pNLn-NanoLuc-Kp and the expression vector for VSV-G using Lipofectamine 2000 Transfection Reagent (Thermo Fisher Scientific). The supernatant was collected and centrifuged to remove the cells two days after transfection. THP-1 cells were infected by being incubated for 24 hours with viral supernatant, washed three times with fresh medium, and cultured for a further 24 hours. Cells were cloned by limiting dilution by seeding at 0.3 cells per well in 96-well cell culture plates. After approximately one month, 232 clones were obtained. Infected clones were identified by assay of NanoLuc activity 24 hours after stimulation with 10 ng/mL phorbol-12-myristate-13-acetate (PMA). NonoLuc activity above background level was observed in 22 out of 232 clones. Furthermore, NanoLuc activity was observed four of these clones at a lower level than the other clones before stimulation with PMA; activity subsequently increased over 20-fold after PMA stimulation. Finally, we selected two clones (#95 and #225) to screen the chemical library for kinase inhibitors. THP-1-NnoLuc clones were seeded at 1.25 × 10^5^ cells per well in 48-well cell culture plates and treated with 0.1% dimethyl sulfoxide (DMSO) only or with kinase inhibitors at dose of 1 or 10 μM for 24 hours. NonoLuc activity was measured after the cells were lysed.

### Measurement of NanoLuc activity

THP-1-NanoLuc clones were seeded at 1.25 × 10^5^ cells per well in 48-well cell culture plates. The cells were incubated with or without SAHA, Tumor necrosis factor-alpha (TNF-α), or PMA for 24 hours, harvested, and lysed in 100 μl of Passive Lysis Buffer (Promega). NanoLuc activity in 5 μl of the lysate was measured using Luciferase Reporter Assay System (Promega).

### Quantitative real-time PCR

The total RNA of the ACH2 and U1 cells was isolated using the SV Total RNA Isolation System (Promega) and reverse transcribed with the PrimeScript RT Master Mix (Takara Bio). Real-time RT-PCR was carried out using SYBR Premix Ex Taq II (Takara Bio) at 95 °C for 30 seconds, followed by 45 cycles of 95 °C for 5 seconds and 60 °C for 30 seconds. The level of *gapdh* expression in each sample was used to standardize the data. The following primer sets were used: HIV-1 *gag* (5′-AGTGGGGGGACATCAAGCAGCCATGCAAAT-3′, 5′-TACTAGTAGTTCCTGCTATGTCACTTCC-3′) and *gapdh* (5′-GCACCGTCAAGGCTGAGAAC-3′, 5′-TGGTGAAGACGCCAGTGGA-3′).

### Measurement of cell viability

Cells were seeded at 2.5 × 10^4^ cells per well in 96-well cell culture plates and treated with 0.1% DMSO only, or with BI-2536 or BI-6727. Cell viability was measured using a Cell Counting Kit-8 (Dojindo Molecular Technologies), according to the manufacturer’s instructions.

### Immunoblotting

ACH2 and U1 cells were seeded at 5 × 10^5^ cells per well in 6-well cell culture plates and untreated (0.1% dimethyl sulfoxide) or treated with 10 ng/mL PMA, 1 μM BI-2536, or 1 μM BI-6727 for 24 h. The cells were harvested and lysed with TNE buffer (10 mM Tris-HCl [pH 7.5], 150 mM NaCl, 1% Nonidet P-40, and 1 mM EDTA). The lysates were separated by SDS-PAGE and transferred to a PVDF membrane (Bio-Rad Laboratories). The membrane was incubated with anti-HIV-1 p24 antibody (ab9044 [Abcam]). Immunoreactive proteins were visualized with anti-mouse IgG conjugated to horseradish peroxidase (MBL) and Amersham ECL Prime Western Blotting Detection Reagent (GE Healthcare) using ImageQuant LAS 500 (GE Healthcare). After detection of HIV-1 p24, the membrane was incubated with striping buffer (62.5 mM Tris-HCl [pH 6.8], 2% SDS, and 100 μM β-mercaptoethanol) and reprobed with anti-alpha-tubulin antibody (Wako Pure Chemical Industries).

### Preparation of PBMCs and quantitative measurements of HIV-1 RNA

We detected cell-associated total HIV-1 mRNA detection method by targeting 3′ LTR region in the previous report^41^. This detecting method is able to measure both Unspliced RNA and Multiply-spiced RNA. We succeeded to detect HIV-1 basal transcription activity in PBMCs as well as lymphocytes obtained by Fine Needle Biopsies of Lymph Nodes obtained from HIV-1 infected individuals. Those patients’ plasma Viral Load (HIV-1 copy in plasma) was suppressed by ART to the levels under limit of detection (<20 copies/mL). Briefly, PBMCs were isolated from 6 mL blood of HIV-1 positive individuals under HAART who had plasma viral RNA loads at less than 20 copies using Ficoll-paque (GE Healthcare) and aliquoted into a 24-well plate using RPMI-1640 medium with 20% fetal bovine serum. For stimulation with JQ1 and BI-2536, PBMCs were expanded by treatment with 20 U/mL IL2, and 1 μg/mL PHA. HIV-1 stimulation was recorded after a 16-hour incubation in a CO_2_ incubator in the presence of BI-2536, JQ1, or PMA. Total RNA was isolated from the cultured PBMCs using a ReliaPrep RNA Mini kit (Promega). HIV-1 transcriptional activity was detected, as described previously^40^, by reverse transcriptase quantitative PCR (RT-qPCR) with PrimeScript RT Master Mix (Takara Bio), using a forward primer (5′-CCA AAG AAG ACA AGA TAT CCT TGA-3′), a reverse primer (5′-TTG AGG CTT AAG CAG TGG G-3′), and a TaqMan probe (FAM-5′-CTG CTT ATA TGC AGC ATC TGA GGG C-BHQ1-3′) at 45 °C for 20 minutes and 95 °C for 30 seconds, followed by 45 cycles of 95 °C for 5 seconds and 60 °C for 30 seconds. HIV-1 transcript levels were normalized HIV-1 copy number per 1 million cells.

This study was approved by the St. Vincent’s Hospital Human Research Ethics Committee (HREC LNR/16/SVH/327). All study participants provided informed consent. All methods were carried out in accordance with the relevant guidelines and regulations.

### Chromatin immunoprecipitation (ChIP)

CHIP analysis was performed using SimpleChIP Plus Enzymatic IP Kit (Cell Signaling Technology) according to the manufacture’s protocol. #95 THP-1-NanoLuc clone cells (4 × 10^6^ cells) were cultured with 0.1% DMSO, 1 μM BI-2536, or 1 μM BI-6727 for 24 hours and fixed in 1% formaldehyde. Cross-linked DNA fragments bound to BRD4 protein were immunoprecipitated using anti-BRD4 antibody (ab128874 [Abcam]). The amount of immunnoprecipitated DNA was determined by quantitative real-time PCR. The following primer sets were used for real-time PCR amplification of the HIV-1 LTR region: 5′-AGTGTGTGCCCGTCTGTTGT-3′, 5′-TTCGCTTTCAGGTCCCTGTT-3′. Results obtained from real-time PCR were normalized by input DNA after signals from DNA fragments imunoprecipitated with normal rabbit IgG (Cell Signaling Technology) were subtracted as a background signal.

### Statistical analysis

In the experiments using cell lines, statistical significance was determined using Student’s *t* test with two-tailed distribution. The Mann-Whitney *U* test was used to analyze unpaired nonparametric data from primary cells.

## Electronic supplementary material

Supplementary Information
