# Supplementary material for: BI-2536 and BI-6727, dual Polo-like kinase/bromodomain inhibitors, effectively reactivate latent HIV-1
Source: Sci Rep. 2018 Feb 23;8:3521. doi: 10.1038/s41598-018-21942-5 (PMC5824842; doi:10.1038/s41598-018-21942-5)
Supplement: Supplementary file 1 — Supplementary Information [file 41598_2018_21942_MOESM1_ESM.pdf]

Supplementary Information

BI-2536 and BI-6727, dual Polo-like kinase / bromodomain inhibitors, effectively reactivate latent HIV-1

Jin Gohda[1,2], Kazuo Suzuki[3], Kai Liu[1], Xialin Xie[1], Hiroaki Takeuchi[4], Jun-ichiro Inoue[2,5], Yasushi Kawaguchi[2,6] & Takaomi Ishida[1,2,*]

[1]China-Japan Joint Laboratory of Molecular Immunology & Microbiology, Institute of Microbiology, Chinese Academy of Sciences, , Beijing, P.R.China
[2]Research Center for Asian Infectious Diseases, The Institute of Medical Science, The University of Tokyo, Tokyo, Japan
[3]Immunovirology Laboratory, St. Vincent's Center for Applied Medical Research, Darlinghurst, New South Wales, Australia
[4]Department of Molecular Virology, Tokyo Medical and Dental University, Tokyo, Japan
[5]Division of Cellular and Molecular Biology, The Institute of Medical Science, The University of Tokyo, Tokyo, Japan
[6]Division of Molecular Virology, The Institute of Medical Science, The University of Tokyo, Tokyo, Japan

*Correspondance should be addressed to T.I. (imoakat@ims.u-tokyo.ac.jp)

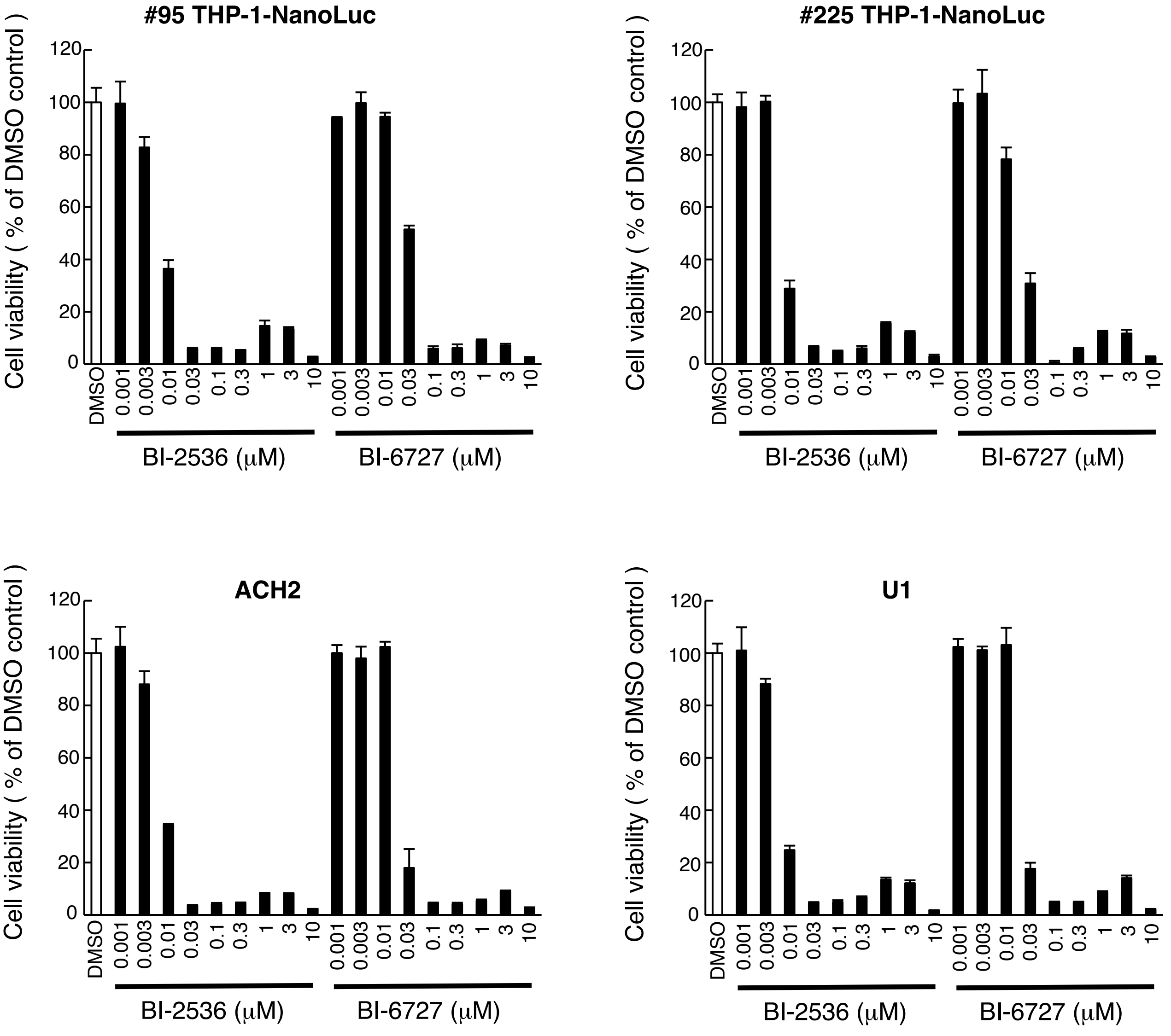

Supplementary Figure 1. Cell viabilities of various cell lines treated with BI-2536 or BI-6727 for 72 hours.

THP-1-NanoLuc clones (#95 and #225), ACH2 and U1 cells were cultured in 0.1% DMSO or in the presence of BI-2536 or BI-6727 at the indicated concentrations for 72 hours. Cell viability was measured using a WST-8 assay and is indicated as a percentage of the viability of DMSO control cells. Each value is shown as a mean ± standard deviation (SD) for triplicate samples.

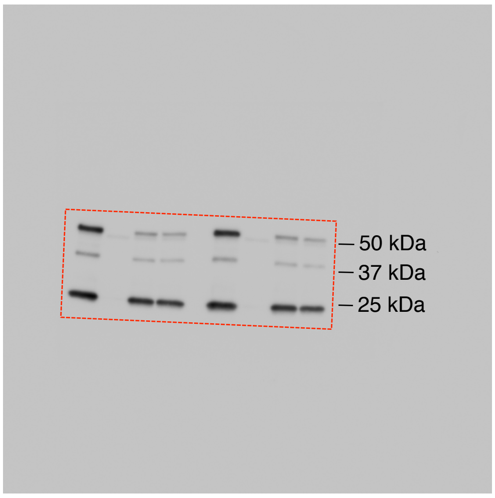

IB: anti-HIV-1 p24

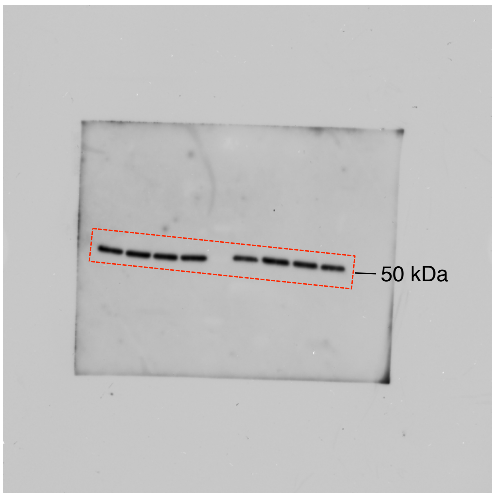

IB: Anti-α-tubulin

Supplementary Figure 2. Uncropped images of immunoblot results shown in Figure 3c. Cell lysates were separated by SDS-PAGE and transferred to a PVDF membrane. The membrane was immunoblotted with anti-HIV-1 p24 antibody (left image). After the antibody on the membrane was stripped, the membrane was immunoblotted with anti-α-tubulin antibody (right). The cropped parts indicated by the boxes with red dashed lines are shown in Figure 3c.

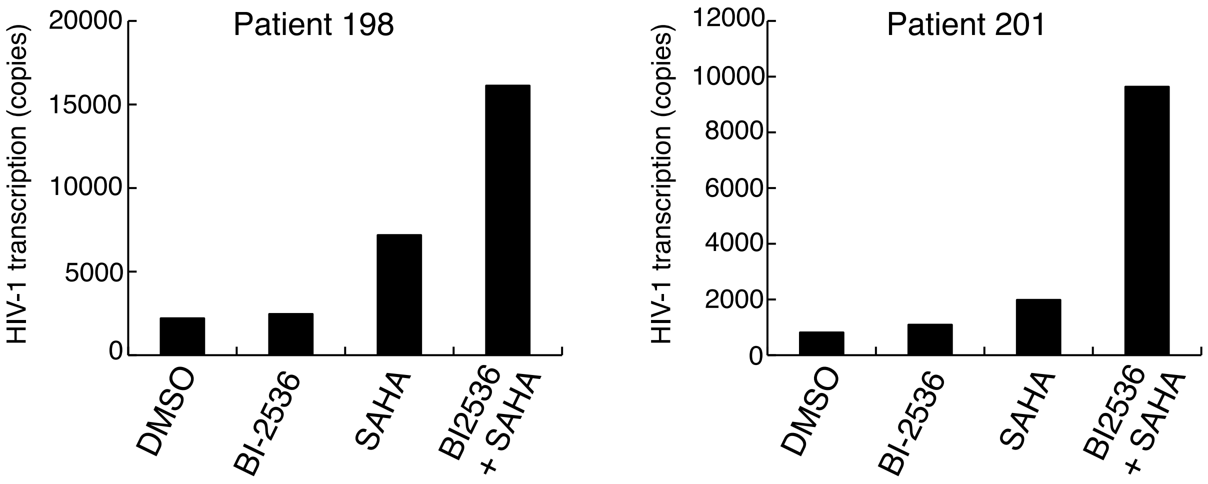

Supplementary Figure 3. Reactivation of latent HIV-1 provirus in primary cells from patients by co-treatment with BI-2536 and SAHA.

Peripheral blood mononuclear cells derived from two different HIV-1 patients were treated with 0.1 μM BI-2536 alone or in combination with 1 μM SAHA for 16 hors. Total RNA was prepared from the cells. *Gag* mRNA expression was measured by quantitative RT-PCR. Viral copy number per one million cells was shown as the mean of triplicate determination.
